# Bibliographical

**Published:** 1883-07

**Authors:** 


### Bibliographical.

WEBB'S "NOTES ON OPERATIVE DENTISTRY."

*Dr. W. C. Barrett, Editor of the Independent Practitioner.*

DEAR SIR :—Certain features of Dr. Webb's book were criticised in your June issue, and the onus for what was considered objectionable was placed upon the publisher. It is only fair to all concerned that the facts should appear.

The agreement to publish these "Notes" was made by letter with Dr. Webb, when he was on his death-bed; made at his request; made on his proposed terms; made for the benefit of his family—his legacy to wife and children; made without question, without modification, and without inspection of the manuscript—indeed, when the matter was perhaps not nearly ready.

The author's copy was scrupulously respected. Every cut in the book was placed as he arranged it in the manuscript, and without hint, suggestion, or request of any kind from the publisher. The volume as it appears is the work of Dr. Webb, and when you say, "To every portion of the book for which Dr. Webb is responsible we can give unhesitating commendation," you cover its contents from title to finis.

Yours respectfully,

THE S. S. WHITE DENTAL M'F'G CO.

[We publish the above according to request, and not because we think there is any necessity for it. The S. S. White Dental M'f'g Co. is quite too large a corporation, and is too well and favorably known, to need any shelter behind the tombstone of Dr. Webb.—EDITOR.]

---

### AN ANOMALY OF THE HUMAN HEART.

Dr. Horace Grant, of Louisville, reports in the July number of the *American Journal of the Medical Sciences* a remarkable anomaly of the human heart, interesting not alone from its striking singularity, but as well from its clinical importance.

In a post-mortem examination of a mulatto girl, aged sixteen years, the right ventricle was found to communicate directly with the aorta ; no pulmonary artery was to be seen attached to the heart. The left auricle was normal, while the left ventricle presented only one-half the usual attachment of the aorta. In a word, both ventricles opened with equal freedom into the aorta. At the pericardial attachment to the aorta two arteries were given off, each about one-fourth of an inch in diameter; they passed right and left backward from the front of the aorta, and evidently supplied the blood to the lungs. This curious anomaly is discussed in connection with the clinical symptoms observed during life.

